# Isolation and characterization of a clade 2.3.4.4b genotype D1.1 H5N1 virus from dairy cattle in Wisconsin

**DOI:** 10.1128/jvi.00761-26

**Published:** 2026-07-10

**Authors:** Daria Mezhenskaia, Lavanya Babujee, Ailam Lim, Lizheng Guan, Dani Nguyen, Chunyang Gu, Gabriele Neumann, Keith Poulsen, Amie J. Eisfeld, Yoshihiro Kawaoka

**Affiliations:** 1 Department of Pathobiological Sciences, Influenza Research Institute, University of Wisconsin-Madison 5228, Madison, Wisconsin, USA; 2 Wisconsin Veterinary Diagnostic Laboratory, University of Wisconsin-Madison 5228, Madison, Wisconsin, USA; 3 Department of Virology, Institute of Medical Science, University of Tokyo 26430, Tokyo, Japan; 4 The University of Tokyo Pandemic Preparedness, Infection and Advanced Research Center (UTOPIA), University of Tokyo, Tokyo, Japan; 5 International Virus Infectious Disease Research Center, National Institute of Global Health and Medicine, Japan Institute for Health Security 739298 , Tokyo, Japan; St Jude Children's Research Hospital, Memphis, Tennessee, USA

**Keywords:** influenza A virus, H5N1, clade 2.3.4.4b, genotype D1.1, dairy cattle, mammalian adaptation, PB2-E627K

## Abstract

Highly pathogenic avian influenza A(H5N1) (HPAI H5N1) viruses of clade 2.3.4.4b have recently been detected in U.S. dairy cattle following multiple spillover events from avian reservoirs. In December 2025, HPAI H5N1 virus was identified in a dairy herd in Wisconsin through the National Milk Testing Strategy. Here, we report the isolation of a clade 2.3.4.4b, genotype D1.1 H5N1 virus, A/dairy cow/Wisconsin/25G05743-001/2025 (WI5743-H5N1), from bulk milk associated with the affected herd, describe its phylogenetic relationships, and assess its pathogenicity in mice. Infectious virus was recovered following blind passage in embryonated chicken eggs. Phylogenetic analysis demonstrated that WI5743-H5N1 is distinct from previously reported D1.1 viruses detected in dairy cattle in Nevada and Arizona, supporting an independent introduction into cattle, and indicating a likely local avian source. Compared with closely related avian viruses, WI5743-H5N1 encoded the mammalian-adapting substitution PB2-E627K and additional amino acid differences in HA, PB1-F2, and NS1. In mice, WI5743-H5N1 replicated efficiently in respiratory tissues and was detectable in the brain but exhibited lower lethality relative to other recent clade 2.3.4.4b, genotype B3.13 viruses. Together, these findings highlight the genetic and phenotypic diversity of HPAI H5N1 viruses infecting dairy cattle and underscore the importance of continued surveillance and functional characterization of emerging strains.

**IMPORTANCE:**

Highly pathogenic avian influenza A(H5N1) viruses have recently entered U.S. dairy cattle through multiple spillover events from avian reservoirs, creating new opportunities for viral adaptation in mammals. Here, we describe the isolation and characterization of a clade 2.3.4.4b, genotype D1.1 H5N1 virus from bulk milk collected during a spillover event in Wisconsin in December 2025. Phylogenetic analyses demonstrated that this virus represents an independent introduction into dairy cattle distinct from previously reported D1.1 viruses identified in Nevada and Arizona. Although the virus encoded the mammalian-adapting PB2-E627K substitution, it exhibited comparatively low lethality in mice, highlighting the complexity of mammalian adaptation and pathogenicity in H5N1 viruses. These findings expand current understanding of the genetic and phenotypic diversity of H5N1 viruses infecting dairy cattle and emphasize the importance of continued surveillance and functional characterization of emerging strains.

## INTRODUCTION

Highly pathogenic avian influenza A(H5N1) viruses (HPAI H5N1) of the A/goose Guangdong/1/1996 lineage first emerged in domestic geese in China in 1996 and subsequently spread widely across Asia, Europe, and Africa (1), with sporadic human infections associated with high case fatality among confirmed cases (2). In late 2021, clade 2.3.4.4b HPAI H5N1 viruses were introduced into the Americas, where they spread rapidly across the continent, became established in wild bird populations, and caused extensive outbreaks in domestic poultry and wild mammals (1, 3–5). In early 2024, clade 2.3.4.4b, genotype B3.13 HPAI H5N1 was first detected in U.S. dairy cattle (6). Additional wildlife-to-cattle spillover events involving clade 2.3.4.4b, genotype D1.1 viruses, identified via the U.S. Department of Agriculture (USDA)’s National Milk Testing Strategy (NMTS) (7), were reported in dairy herds in Nevada and Arizona in early 2025 (8, 9). To date, more than 1,000 U.S. dairy herds have been affected (6), with documented interspecies transmission to poultry, domestic cats, and humans (10).

In December 2025, a fourth spillover event involving a clade 2.3.4.4b, genotype D1.1 virus was confirmed in Dodge County, Wisconsin, through the USDA NMTS (11–13). No clinical illness or decrease in milk production was observed in the affected herd, and infection was estimated to have occurred several weeks prior to detection based on viral RNA levels and serologic findings. No additional infected herds were identified in the surrounding region, despite the high density of dairy operations in Dodge County (133 dairies) and neighboring counties (515 dairies). The affected farm is near major waterfowl habitats, including Horicon Marsh, a 33-square-mile state and national wildlife refuge, and Fox Lake (~2,700 acres), consistent with a potential wildlife source of introduction. Here, we report the isolation of an HPAI H5N1 virus from bulk milk associated with the affected dairy herd, phylogenetic analysis of its genome sequence, and evaluation of its pathogenicity in mice.

## MATERIALS AND METHODS

### Biosafety

Experiments with HPAI H5N1 viruses were carried out in biosafety level 3 (BSL-3) containment laboratories at the Influenza Research Institute at the University of Wisconsin–Madison, which is approved by the Federal Select Agent Program for studies with these viruses. Funding for this study came in part from the NIAID Centers of Excellence for Influenza Research and Response (CEIRR, Contract Number 75N93021C00014). All experiments were approved by the Institutional Biosafety Committee of the University of Wisconsin–Madison and all animal experiments were approved by the University of Wisconsin-Madison School of Veterinary Medicine Institutional Animal Care and Use Committee. The NIAID grant for the studies conducted was reviewed by the University of Wisconsin–Madison Dual Use Research of Concern (DURC) Subcommittee in accordance with the 2024 United States Government Policy for Oversight of Dual Use Research of Concern and Pathogens with Enhanced Pandemic Potential and determined not to meet the criteria of DURC or Pathogens with Enhanced Pandemic Potential (PEPP). The University of Wisconsin–Madison Institutional Contact for Dual Use Research reviewed this manuscript and confirmed that the studies described herein do not meet the criteria of DURC.

### Specimen

A fresh bulk milk sample was collected from the affected farm 1 day after the initial detection of HPAI through NMTS surveillance. The sample was submitted the same day to the Wisconsin Veterinary Diagnostic Laboratory, where HPAI H5 clade 2.3.4.4b positivity was confirmed by PCR and antibodies against influenza A virus were detected by ELISA, as previously described (14). The sample was subsequently stored frozen, and an aliquot of the bulk milk sample was provided to the University of Wisconsin–Madison Influenza Research Institute for further virologic characterization.

### Chicken eggs

Specific-pathogen-free (SPF) embryonated chicken eggs (Valo BioMedia) were grown at 37°C with appropriate humidity. Ten-day-old eggs were candled to verify embryo viability prior to inoculation. Following inoculation, eggs were incubated at 35°C with appropriate humidity.

### Cells

Madin-Darby canine kidney (MDCK) cells were grown in Eagle’s minimal essential medium (MEM) containing 5% newborn calf serum and antibiotics. The incubation conditions for cell growth, virus growth, and plaque assays were 37°C in a humidified atmosphere of 5% CO_2_. The cells were routinely monitored for mycoplasma contamination.

### Egg and cell inoculation

Whole milk was centrifuged at 6,800 × *g* and the skim fraction was collected and mixed with 100× antibiotic-antimycotic solution (“Anti-Anti,” Gibco) at a ratio of 1:10. The sample was then serially diluted (10^−1^ through 10^−5^) in 1× phosphate-buffered saline (PBS) and used to inoculate SPF eggs (10^0^ through 10^−2^ dilutions, 250 μL per egg, 2 eggs per dilution) or MDCK cells in a six-well plate (10^0^ through 10^−5^ dilutions, 500 μL per well, one well per dilution). Inocula were removed from MDCK cells after 1 h of incubation, the cells were washed once with PBS, and then cells were covered with MEM containing 0.3% bovine serum albumin (BSA), 0.6 μg/mL of L-(tosylamido-2-phenyl) ethyl chloromethyl ketone (TPCK)-treated trypsin, and antibiotics. Inoculated eggs were incubated for 72 h, candled to assess viability, and then chilled at 4°C overnight. Inoculated MDCK cells were incubated for 5 days and observed daily by microscopy to assess cytopathic effects. For subsequent passages in eggs, allantoic fluids were collected from previously inoculated eggs and centrifuged at 1,300 × *g* to remove debris. Clarified allantoic fluids were then mixed with Anti-Anti at a ratio of 1:5, used to infect fresh SPF eggs without further dilution (200 μL per egg in each of five to six eggs), and eggs were incubated for 36–72 h before candling and chilling at 4°C as described above. For subsequent passages in MDCK cells, cell culture medium was collected from the 10^0^ through 10^−2^ dilutions, centrifuged at 1,300 × *g* to remove debris, and then used to infect fresh MDCK cell monolayers in six-well plates without further dilution (500 μL per well, two wells per dilution). After three passages in eggs, a high titer virus stock was obtained, aliquoted, and frozen at –80°C.

### Hemagglutination assays

Hemagglutination assays were carried out using standard methods. Briefly, allantoic fluids from eggs or MDCK cell culture supernatants were twofold serially diluted and mixed with 0.5% turkey red blood cells, followed by assessment for agglutination.

### Egg infectious dose 50 analysis (EID_50_)

The egg virus stock was thawed and serially diluted (10-fold, through 10^−13^) in PBS. SPF eggs were then inoculated with 200 μL of the 10^−7^ through 10^−13^ dilutions (three eggs per dilution) and incubated at 35°C with humidity for 48 h before candling and chilling as described. Allantoic fluids were tested by HA assays (as described), and the EID_50_ was calculated according to the method of Reed and Muench (15).

### Sequencing

RNA was extracted from the egg virus stock by using the QIAamp viral RNA mini kit (Qiagen) according to the manufacturer’s instructions. Reverse transcriptase polymerase chain reaction (RT-PCR) assays were carried out with the SuperScript III One-Step RT-PCR System (Invitrogen) and universal primers (primer sequences are available upon request) according to the manufacturer’s instructions. Amplification of gene segments was confirmed by agarose gel electrophoresis, and then the segments were individually sequenced with Oxford Nanopore Technologies sequencing at the University of Wisconsin-Madison Biotechnology Center.

### Phylogenetic analysis

Phylogenetic analysis of D1.1 viruses was performed using concatenated whole-genome consensus sequences. Sequences were either generated in-house or retrieved from publicly available data sets (Andersen Lab, https://github.com/andersen-lab/avian-influenza; accessed 30 January 2026) and aligned using MAFFT (v7.490) (16). Maximum likelihood phylogenies were inferred with IQ-TREE2 (17), utilizing a GTR+F substitution model and 1,000 ultrafast bootstrap replicates. The tree was rooted using A/Western gull/CA/25-002655-001/2024 as an outgroup and visualized by using the ggtree package in R (18).

### Mouse lethal dose 50 (MLD_50_)

Seven-week-old female BALB/cJ mice (RRID:IMSR_JAX:000651, The Jackson Laboratory) were randomly assigned to experimental groups, deeply anesthetized by isoflurane inhalation, and intranasally infected with serial dilutions (10^0^, 10^1^, 10^2^, 10^3^, 10^4^, 10^5^, or 10^6^ PFU) of WI5743-H5N1 in 50 µL of PBS (five mice per dose). Body weights and survival were monitored daily, and animals were euthanized if they lost ≥35% of their initial body weight or were unable to remain upright. The MLD_50_ was calculated according to the method of Reed and Muench (15).

### Tissue tropism in mice

Seven-week-old female BALB/cJ mice (RRID:IMSR_JAX:000651, The Jackson Laboratory) were randomly assigned to experimental groups, deeply anesthetized by isoflurane inhalation, and intranasally infected with 10^3^ PFU of WI5743-H5N1 in 50 µL of PBS. Groups of five mice were euthanized on days 3 and 6 post-infection, and brain, nasal turbinate, and lung tissues were collected using instruments that were disinfected between each tissue to prevent cross-contamination. Tissues were directly frozen at –80°C without buffer.

### Plaque assays

Allantoic fluids and MDCK cell culture supernatants were used directly in plaque assays with or without prior freezing. Frozen mouse tissues were thawed, homogenized in 1 mL of 1× MEM containing 0.3% BSA with a TissueLyser II (Qiagen) (30 Hz oscillation frequency for 3 min), clarified by centrifugation (20,000 × *g* for 10 min), and the supernatants were used for plaque assays. Plaque assays were performed in MDCK cells using standard methods. Briefly, MDCK cells were washed with 1× MEM containing 0.3% BSA and inoculated with serial dilutions of allantoic fluids, MDCK cell culture supernatants, or clarified tissue homogenates. Inocula were removed after 1 h incubation, and then cells were washed with 1× PBS and overlaid with 1× MEM containing 0.3% BSA, 1% low melting point agarose, and 0.6 µg/mL TPCK-treated trypsin. After 2–3 days of incubation, the overlaid cells were fixed with 10% formalin, agarose overlays were removed, and plates were rinsed with water and air-dried. Virus plaques were counted under fluorescent light.

### Statistics and reproducibility

All animals were randomly allocated to experimental groups. No blinding was performed in any experiment. Sample sizes were based on our previous work. All experiments with animals were performed once. All graphs were prepared with GraphPad Prism software, version 9.5.1.

## RESULTS

### Virus isolation from bulk milk

A bulk milk sample was collected from the affected farm 1 day after initial detection of HPAI through NMTS surveillance. Testing at the Wisconsin Veterinary Diagnostic Laboratory confirmed the presence of HPAI H5 clade 2.3.4.4b viral RNA by multiple PCR assays (Ct values ranging from 30.9 to 33.7) and antibodies against influenza A virus were detected by ELISA (*S*/*N* ratio = 0.49). Subsequently, the bulk milk was inoculated into MDCK cells or 10-day-old embryonated chicken eggs to attempt virus isolation. No cytopathic effects or embryo mortality were observed, and no virus was detected by hemagglutination (HA) assay in allantoic fluid or cell culture supernatants from passages 1 (P1) or 2 (P2) (Table 1). In passage 3 (P3), four of six chicken embryos died, and allantoic fluid exhibited an HA titer ≥2,048. In contrast, MDCK cells showed only minimal cytopathic effects, and no virus was detected by HA assay (Table 1). Plaque formation in MDCK cells was observed for allantoic fluids from egg P2 and P3 and for cell culture supernatant from MDCK P3, with substantially higher titers in egg-derived samples (e.g., 3.8 × 10^7^ PFU/mL in egg P3 compared to 500 PFU/mL in MDCK P3; Table 1). Because P3 allantoic fluid collected at 72 h post-infection appeared turbid, the P2-to-P3 passage was repeated with earlier collection at 36 h post-infection (designated P3b). In this experiment, all chicken embryos died, and allantoic fluid was clear, with an HA titer of 2,048 and a plaque assay titer of 6 × 10^10^ PFU/mL (Table 1). Egg infectious dose 50 (EID_50_) titrations of P3b, performed in duplicate, yielded values of 1.6 × 10^10^ and 1.0 × 10^10^. Collectively, these data demonstrate successful isolation of an influenza virus from bulk milk collected at a Wisconsin dairy farm (A/dairy cow/Wisconsin/25G05743-001/2025 [H5N1]; hereafter WI5743-H5N1).

**TABLE 1:** Virus isolation from bulk milk^*a*^

Passage	Growth substrate	Viability	HA titer	PFU/mL	Fluid color
P1	Eggs	100%	Negative	n.d.^*c*^	Clear
P2	Eggs	100%	Negative	<10^4^	Slightly cloudy
P3	Eggs	33%	≥2,048	3.8 × 10^7^	Turbid
P3b^*b*^	Eggs	0%	2,048	6.0 × 10^10^	Clear
P1	MDCK	100%	Negative	n.d.	Normal
P2	MDCK	100%	Negative	n.d.	Normal
P3	MDCK	>90%	Negative	500	Normal

^*a*^ Serial dilutions of bulk milk were passaged in embryonated chicken eggs or in MDCK cells. Characteristics of each passage in each growth substrate are given.

^*b*^ P3 and P3b were generated similarly in embryonated chicken eggs but differed in harvest time, with P3 collected at 72 h (turbid fluid) and P3b at 36 h (clear fluid).

^*c*^ n.d., not determined.

### Phylogenetic and sequence analysis

**Fig 1:**
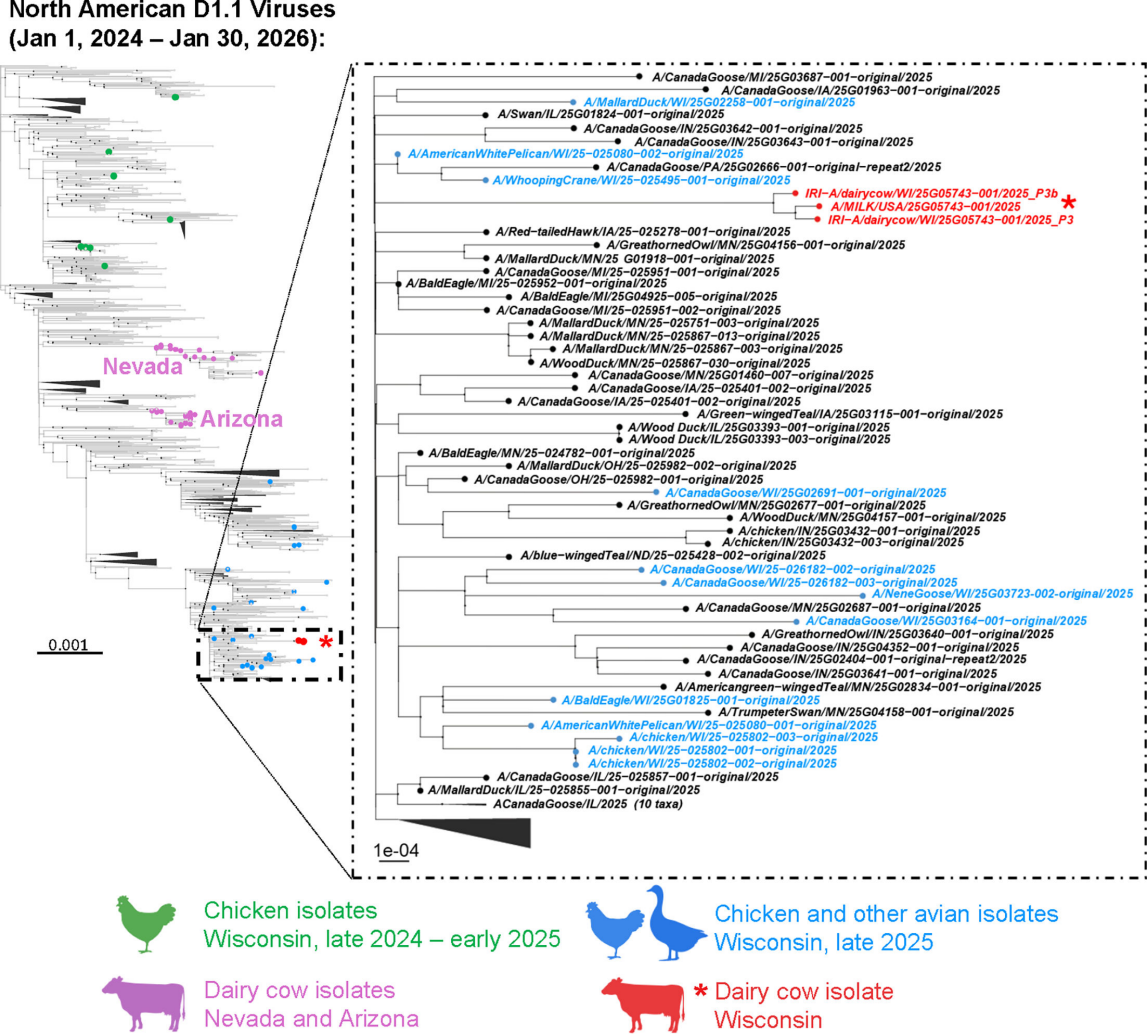
Phylogenetic analysis of WI5743-H5N1. Concatenated nucleotide sequences of all unique North American clade 2.3.4.4b, genotype D1.1 viruses from 1 January 2024, through 30 January 2026, along with the sequence of the WI5743-H5N1 virus isolate generated in this study (indicated in the tree by the prefix “IRI”), were used for phylogenetic analysis. The full phylogenetic tree is shown on the left, and a zoomed-in segment of the tree is shown at the right. A color key is given at the bottom.

RNA extracted from P3 and P3b allantoic fluids was subjected to deep sequencing, which identified WI5743-H5N1 as an HPAI H5N1 virus belonging to clade 2.3.4.4b, genotype D1.1. Phylogenetic analysis showed that WI5743-H5N1 did not cluster with genotype D1.1 viruses previously isolated from infected dairy cattle in Nevada and Arizona (Fig. 1), consistent with a report from the USDA Animal and Plant Health Inspection Service (APHIS) describing the Wisconsin detection as a separate wildlife-to-cattle spillover event (12) and supporting an independent introduction of D1.1 viruses into cattle. WI5743-H5N1 was closely related to genotype D1.1 viruses isolated from domestic chickens and multiple wild avian species in Wisconsin in late 2025 (Fig. 1), indicating a likely local avian source. Compared with these closely related avian viruses, WI5743-H5N1 encoded the mammalian-adapting substitution PB2-E627K, which has been previously associated with enhanced replication and virulence of avian influenza viruses in mammalian hosts (19, 20). Notably, PB2-E627K has also been identified in at least one recent avian D1.1 virus, A/peafowl/USA/25G05091-001/2025, indicating that this substitution is not restricted to mammalian isolates. Additional amino acid differences between WI5743-H5N1 and its closest avian relatives included HA-P321H (numbering based on mature H5 HA), PB1-F2-C57Y, and NS1-Y218Stop. The introduction of a stop codon at NS1 residue 218 results in a truncated NS1 protein, which may alter its antagonism of host antiviral responses. The functional significance of the HA-P321H and PB1-F2-C57Y substitutions is currently unknown.

Sequence comparisons with data generated by the USDA APHIS (for RNA isolated from the bulk milk sample: A/milk/USA/25G05743-001/2025) revealed minor differences at two positions. At NA residue 369, located within the second sialic acid binding site (but not considered essential for receptor interaction [21]), APHIS reported serine. In our egg P3 virus, arginine was detected at this position, whereas in egg P3b both serine and arginine were present at frequencies of 88% and 12%, respectively. At M1 residue 83, APHIS reported a mixed population of threonine and alanine, whereas threonine was detected in both egg P3 and P3b viruses. The functional significance of this substitution is unknown.

### Mouse lethal dose 50 (MLD_50_)

**Fig 2:**
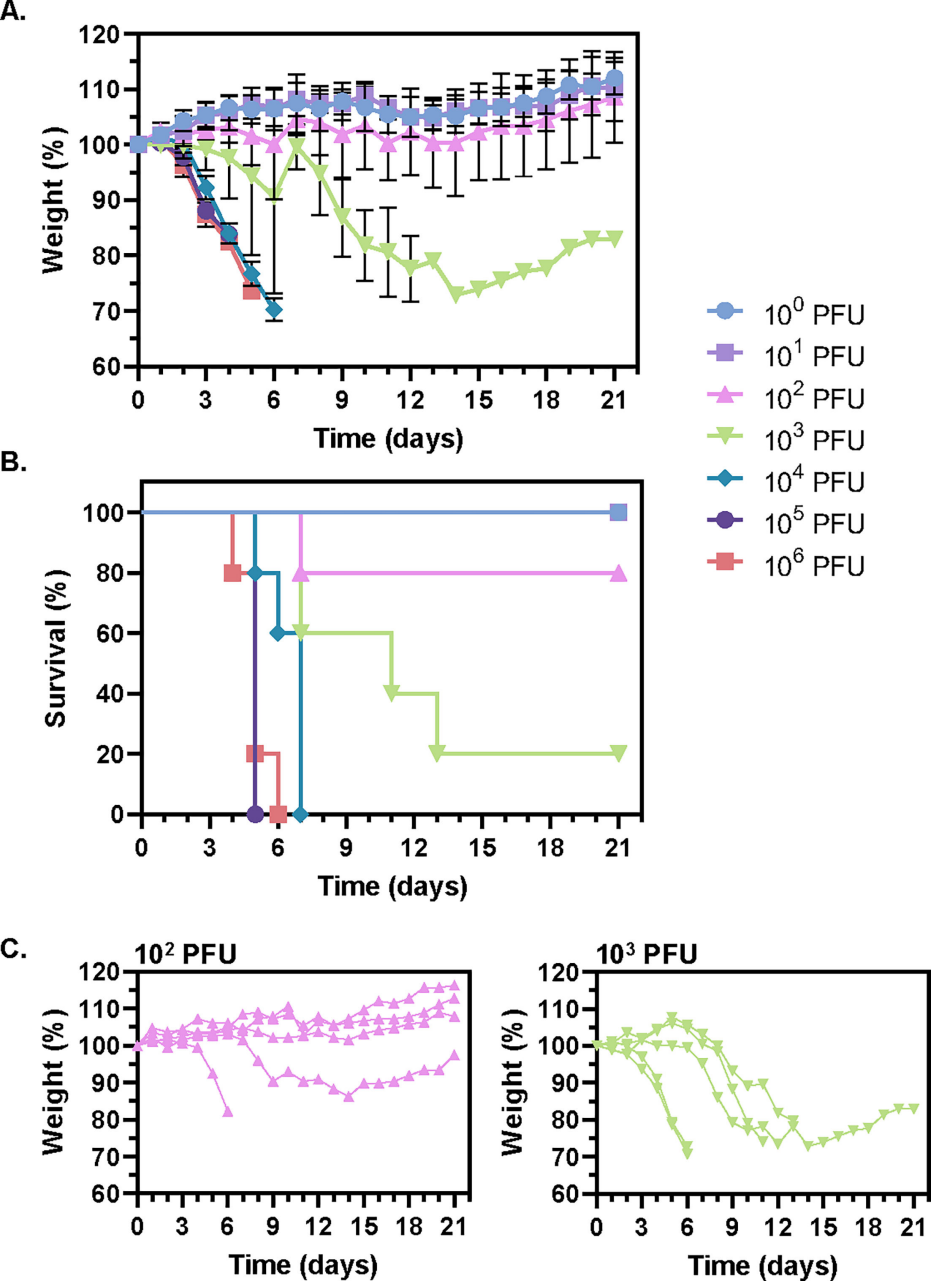
Mouse lethal dose 50 analysis. Female BALB/cJ mice (7 weeks old, *n* = 5 biologically independent animals per dosage) were deeply anesthetized and intranasally inoculated with 10-fold serial dilutions of WI5743-H5N1 in 50 μL of PBS. (**A**) Body weight and (**B**) survival were monitored daily for 21 days. In panel A, the error bars represent the standard deviation. (**C**) Individual body weights are shown for animals infected with 10^2^ (left) or 10^3^ (right) PFU.

Since the WI5743-H5N1 virus encodes the mammalian-adapting substitution, PB2-627K, we evaluated its pathogenicity in the mouse model by using a mouse lethal dose 50 (MLD_50_) assay. Groups of female BALB/cJ mice (*n* = 5 per dose) were intranasally inoculated with 10-fold serial dilutions of WI5743-H5N1 in PBS (10^0^–10^6^ PFU), and body weight and survival were monitored daily for 21 days. Mice infected with 10^0^–10^1^ PFU remained asymptomatic, whereas those infected with ≥10^4^ PFU exhibited rapid weight loss (Fig. 2A) and died by day 6 (Fig. 2B). Intermediate doses (10^2^–10^3^ PFU) resulted in partial lethality (Fig. 2C). The calculated MLD_50_ was 316 PFU. This value is approximately 10-fold higher than that reported for a clade 2.3.4.4b, genotype B3.13 dairy cattle isolate (A/dairy cattle/New Mexico/A240920343-93/2024; MLD_50_ = 31.6 PFU) (22), which lacks PB2-627K but encodes another mammalian-adapting mutation, PB2-631L; and substantially higher than that of a clade 2.3.4.4b, genotype B3.13 human isolate encoding PB2-627K (A/Texas/37/2024; MLD_50_ <1 PFU) (23). Together, these data indicate that WI5743-H5N1 is less lethal in mice than other recent clade 2.3.4.4b viruses, despite encoding the mammalian-adapting PB2-627K substitution.

### Virus replication in respiratory tissues and brain

**Fig 3:**
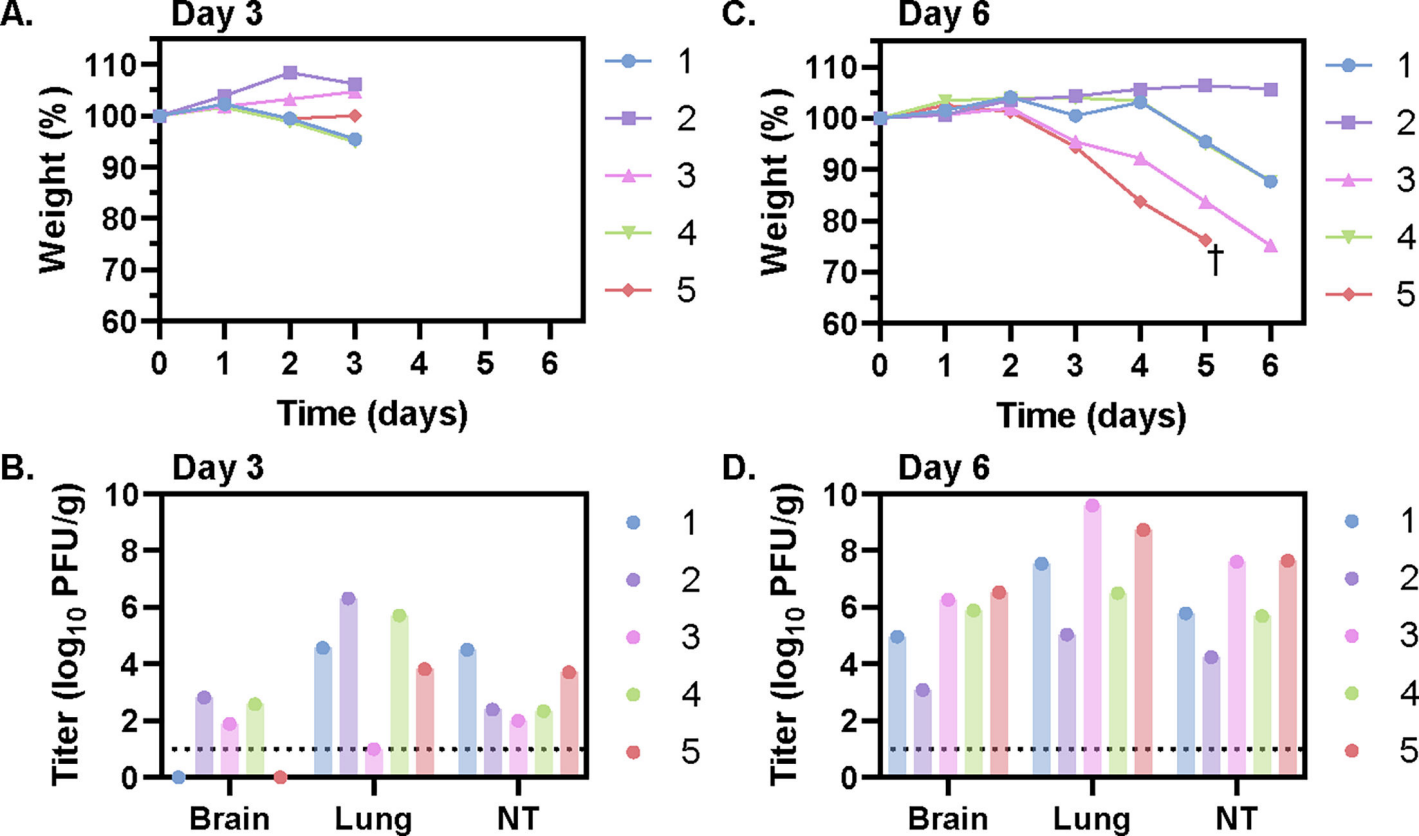
Tissue tropism. Female BALB/cJ mice (7 weeks old, *n* = 5 biologically independent animals per time point) were deeply anesthetized and intranasally inoculated with 10^3^ PFU of WI5743-H5N1 in 50 μL of PBS. Mice were euthanized at day 3 (**A and B**) or day 6 (**C and D**) post-infection, and brain, nasal turbinate (NT), and lung tissues were collected for plaque assays in MDCK cells. Body weights are given in panels A and C, while tissue titers are shown in panels B and D. Color keys for individual mice (1–5 at each time point) are shown to the right of each panel. † indicates an animal that succumbed prior to the designated time point.

We next evaluated replication of WI5743-H5N1 virus in mouse respiratory and brain tissues. Groups of female BALB/cJ mice (*n* = 5 per time point) were intranasally inoculated with 10^3^ PFU of WI5743-H5N1 in PBS, and nasal turbinate, lung, and brain tissues were collected at days 3 and 6 post-infection for virus titration by plaque assays in MDCK cells (Fig. 3). At day 3 post-infection, mice exhibited little to no body weight loss (Fig. 3A). Virus was detected in the nasal turbinates and lungs of all five mice and in the brains of three of five mice, with highest titers observed in lung tissue (Fig. 3B). By day 6 post-infection, body weight loss was evident in four of five mice (Fig. 3C), and virus was detected in all three tissues of all animals (Fig. 3D). Virus titers were generally higher on day 6 than at day 3, with the highest titers observed in lung tissue at both time points (10^6.3^ on day 3 and 10^9.6^ on day 6; Fig. 3B and D). Collectively, these data indicate that WI5743-H5N1 replicates efficiently in both the upper and lower respiratory tracts and is capable of spreading to non-respiratory tissues in mice.

## DISCUSSION

In this study, we report the isolation of a clade 2.3.4.4b, genotype D1.1 HPAI H5N1 virus (WI5743-H5N1) from bulk milk collected in Wisconsin in December 2025 and describe its phylogenetic relationships and pathogenicity in mice. Phylogenetic analyses demonstrated that WI5743-H5N1 is distinct from previously described D1.1 viruses detected in dairy cattle in Nevada and Arizona, supporting an independent introduction into cattle and reinforcing the conclusion that genotype D1.1 viruses have entered dairy cattle through multiple, separate spillover events. The close relationship of WI5743-H5N1 to viruses circulating in domestic and wild birds in Wisconsin further supports a local avian source for this introduction. In a mouse model, WI5743-H5N1 replicated efficiently in respiratory tissues and was detectable in the brain, but exhibited comparatively low lethality (relative to other clade 2.3.4.4b viruses examined elsewhere [22, 23]), despite encoding the mammalian-adapting PB2-627K substitution. Together, these findings highlight the genetic and phenotypic diversity of HPAI H5N1 viruses infecting dairy cattle and underscore the importance of continued surveillance and functional characterization of emerging strains.

Mammalian adaptation of avian influenza A viruses is frequently associated with amino acid substitutions in the PB2 polymerase protein, including E627K, D701N, and M631L (19, 20, 24, 25). These mutations enhance viral replication in mammalian hosts by affecting viral polymerase activity or nuclear import of the viral ribonucleoprotein complex. In the context of HPAI H5N1 viruses detected in dairy cattle, different spillover events have been associated with distinct PB2 substitutions: while WI5743-H5N1 encodes PB2-E627K, the Nevada D1.1 viruses encode PB2-D701N (26), most B3.13 dairy cattle viruses encode PB2-M631L, and some B3.13 dairy cattle viruses encode both PB2-E627K and PB2-M631L (sequences are available at GISAID.org). These observations support the idea that multiple evolutionary pathways can facilitate adaptation of avian influenza viruses to mammalian hosts. However, the functional consequences of these substitutions, particularly across different viral genetic backgrounds, are not fully defined. Although PB2-E627K has been associated with increased virulence in mammalian models, the comparatively low lethality of WI5743-H5N1 suggests that PB2-E627K alone is insufficient to confer a highly virulent phenotype and that additional viral determinants contribute to pathogenicity. Additional experiments are required to more fully clarify how the genetic background supports or constrains the activity of this substitution. Notably, PB2-E627K has also been identified in at least one recent avian D1.1 virus (A/peafowl/USA/25G05091-001/2025), raising the possibility that this substitution may be present in avian viruses prior to spillover into mammals.

The bulk milk sample analyzed in this study was positive for both HPAI H5N1 viral RNA and antibodies against influenza A virus. The presence of antibodies in bulk milk may reduce the efficiency of virus isolation through neutralization, even when viral RNA levels are high. Here, infectious virus was recovered after two serial blind passages in embryonated chicken eggs, suggesting that this approach may help overcome antibody-mediated inhibition and facilitate virus isolation from samples with high antibody levels.

In summary, WI5743-H5N1 represents a distinct D1.1 virus, introduced into dairy cattle through an independent spillover event, that exhibits unique genetic features and phenotypic properties in mammals. The diversity observed among HPAI H5N1 viruses infecting dairy cattle, including differences in phylogenetic origin, adaptive mutations, and virulence, underscores the importance of continued genomic and functional studies to better understand the determinants of host adaptation and pathogenicity.

## Data Availability

All source data described herein are available upon request. Consensus sequences of the WI5743-H5N1 virus isolates have been deposited in NCBI GenBank. WI5743-H5N1_P3 accessions include PZ350406, PZ350407, PZ350408, PZ350409, PZ350410, PZ350411, PZ350412, and PZ350413. WI5743-H5N1_P3b accessions include PZ350414, PZ350415, PZ350416, PZ350417, PZ350418, PZ350419, PZ350420, and PZ350421.
